# Stress and body condition are associated with climate and demography in Asian elephants

**DOI:** 10.1093/conphys/cov030

**Published:** 2015-06-30

**Authors:** Hannah S Mumby, Khyne U Mar, Chatchote Thitaram, Alexandre Courtiol, Patcharapa Towiboon, Zaw Min-Oo, Ye Htut-Aung, Janine L Brown, Virpi Lummaa

**Affiliations:** af1 Department of Animal and Plant Sciences, University of Sheffield, Sheffield S10 2TN, UK; af2 Faculty of Veterinary Medicine, Chiang Mai University, Chiang Mai 50100, Thailand; af3 Department of Evolutionary Genetics, Leibniz Institute for Zoo and Wildlife Research, Alfred-Kowalke-Straße 17 10315 Berlin, Germany; af4 Ministry of Environmental Conservation and Forestry, Myanma Timber Enterprise, Yangon, Myanmar; af5 Department of Veterinary Medicine, Yezin University, Myanmar; af6 Center for Species Survival, Smithsonian Conservation Biology Institute, Front Royal, VA 22630, USA

**Keywords:** *Elephas maximus*, faeces, glucocorticoids, hormone, seasonality, weight

## Abstract

Establishing links between ecological variation, physiological markers of stress and demography is crucial for understanding how and why changes in environmental conditions affect population dynamics, and may also play a key role for conservation efforts of endangered species. However, detailed longitudinal studies of long-lived species are rarely available. We test how two markers of stress and body condition vary through the year and are associated with climatic conditions and large-scale mortality and fertility variation in the world's largest semi-captive population of Asian elephants employed in the timber industry in Myanmar. Glucocorticoid metabolites (used as a proxy for stress levels in 75 elephants) and body weight (used as a proxy for condition in 116 elephants) were monitored monthly across a typical monsoon cycle and compared with birth and death patterns of the entire elephant population over half a century (*n* = 2350). Our results show seasonal variation in both markers of stress and condition. In addition, this variation is correlated with population-level demographic variables. Weight is inversely correlated with population mortality rates 1 month later, and glucocorticoid metabolites are negatively associated with birth rates. Weight shows a highly positive correlation with rainfall 1 month earlier. Determining the factors associated with demography may be key to species conservation by providing information about the correlates of mortality and fertility patterns. The unsustainability of the studied captive population has meant that wild elephants have been captured and tamed for work. By elucidating the correlates of demography in captive elephants, our results offer management solutions that could reduce the pressure on the wild elephant population in Myanmar.

## Teaser

Levels of “stress” hormones and body weight vary seasonally in Asian elephants, with both peaking in the months with heavy workload, best resource availability and lowest birth rates. Our results suggest that a range of both anthropogenic and natural seasonal factors may affect demography in semi-captive populations.

## Introduction

Understanding how ecological variation relates to demography, reproductive ability and underlying physiological correlates of stress and body condition is central for predicting population dynamics and thus for the conservation of endangered species (Hing *et al.*, 2014). Longitudinal population studies based on individual data on such links are rare, and particularly difficult to obtain for species with slow life histories. The definition of stress is sometimes poorly described, but here we refer to it as homeostasis being disrupted by exposure to an event or force known as a stressor (Reeder and Kramer, 2005). In ecological studies, measures of hormones associated with stress response, particularly corticosteroids, are often used as markers of stress (Madliger and Love, 2014). While this approach has limitations, there is some evidence that on a yearly or short-term basis, variation in markers of stress is associated with reproduction and survival in a range of species (Crespi *et al.*, 2013). For example, a study of long-lived black-legged kittiwakes (*Rissa tridactyla*) measured productivity (total number of chicks produced divided by the number of nest attempts) and found that corticosterone levels of breeding individuals in the year with highest productivity were less than half of those in the 2 years with lowest production (Buck *et al.*, 2007). Similar effects of stress levels (as measured by baseline plasma corticosterone or faecal glucocorticoid metabolites) on fitness outcomes, such as fledging success, have also been found in shorter-lived species of mammals (Cabezas *et al.*, 2007), passerine birds (Bonier *et al.*, 2009b) and reptiles (Bonier *et al.*, 2009a). However, the associations are by no means conclusive, with some findings indicating a positive association between corticosterone and markers of fitness, such as chick mass (Crossin *et al.*, 2012), and other studies finding no association between corticosterone and fitness (reviewed by Bonier *et al*., 2009a). Furthermore, most of these studies are conducted on a short-term individual basis and do not consider implications for long-term population-level patterns.

Body weight is an indicator of body condition for longitudinally monitored individuals, and variation can indicate changes in health status (Hile *et al.*, 1997). Weight changes in non-pregnant, determinately growing species represent changes in condition, and the overall weight is also a marker of past condition because larger body size is indicative of good early life conditions and high investment in body growth as opposed to or in addition to immune function or repair (Bateson *et al.*, 2004). It is widely assumed that body condition is inversely associated with markers of stress, and some studies do support this, including those on wild birds (Kitaysky *et al.*, 1999; Cockrem *et al.*, 2006) and some mammals, such as badgers (*Meles meles*; George *et al.*, 2014) and Weddell seals (*Leptonychotes weddellii*; Bartsh *et al.*, 1992). In contrast, a study on degus (*Octodon degus*) found that stress-induced cortisol was positively correlated with body weight in females, but not males (Bauer *et al.*, 2013). Therefore more studies are required to analyse the relationship between body weight and measures of cortisol.

Here, we analyse how seasonal variation in ‘stress’ [as measured by faecal glucocorticoid metabolite (GCM) concentration] and body condition (as measured by body weight) are associated with long-term population-level variation in demographic parameters in Asian elephants (*Elephas maximus*). Glucocorticoid metabolites from faecal matter are used as a non-invasive approach to measuring cortisol, a hormone released in response to stressors as part of the normal physiological function of mammals, including Asian elephants (Menargues *et al.*, 2012). Across species, consistently high GCM concentrations are indicative of breakdown of a regulatory feedback loop (Dallman, 2010). This results in chronic stress, which is also manifested in animals through effects on weight, behaviour, reproduction and immune function (Buckingham, 2010). Therefore, GCM concentrations measured through the year can be used as an objective indicator of welfare (as a marker of stress and health; Mason and Veasey, 2010).

Asian elephants are large, aseasonally breeding mammals (Fowler and Mikota, 2006) that inhabit regions with seasonally fluctuating conditions (Mumby *et al.*, 2013b). They are endangered across their distribution (IUCN, 2014), with Myanmar maintaining the world's largest remaining semi-captive population of almost 5000 elephants (Mar *et al.*, 2012) and the second largest overall population after India with an additional 5000 or fewer wild elephants (Sukumar, 2006). Over half of this semi-captive population is owned by the Myanmar Government and employed in the timber industry (Mar, 2007). In common with most other Asian elephant populations worldwide, the semi-captive Myanmar population is currently unsustainable owing to high infant mortality and low birth rate (Leimgruber *et al.*, 2008). Being a large population living in its original forest environment, it presents a rare example of a potentially sustainable pool of Asian elephants (Sukumar, 2006). There is considerable time pressure to increase reproductive and survival rates because the semi-captive population is currently supplemented with wild-caught elephants, a practice predicted to contribute to the extinction of the wild population within 100 years (Leimgruber *et al.*, 2008). Determining the physiological markers associated with mortality and reproduction is key to maintaining the population of semi-captive elephants and protecting the remaining wild population.

In most of the range area of Asian elephants, climate follows a tropical monsoon pattern within each year. The strict work schedule of the timber elephants, with different work tasks undertaken at different times of the year, adds a further level of variability to the highly seasonal environment (Toke Gale, 1974; Zaw, 1997). Elephants respond to the seasonal environment with a peak in conception rates during the annual rest period, which begins in February and ends with the commencement of work in June (Mumby *et al.*, 2013a), based on a gestation duration of 22 months (Lueders *et al.*, 2012). Survival patterns are also influenced by seasonal conditions, with highest survival rates occurring in months with high rainfall and intermediate temperatures (∼24°C; Mumby *et al.*, 2013b). However, the underlying proximate causes and physiological correlates of these associations are unknown.

Understanding how hormonal and body condition variation by season underlies population-level demographic patterns (fertility and survival rates) is important for intervention strategies, but testing this prediction has not been possible due to the rarity of data sets matching long-term data on population demography, climate and individual-based physiological markers. However, there is some previous evidence to suggest that Asian elephants in their natural range areas exhibit seasonal variation in reproductive hormone levels (Thitaram *et al.*, 2008), with a prolonged luteinizing phase of the ovulatory cycle in the monsoon season. Outside their range areas and in zoo settings, there is also evidence for seasonal variation in GCM concentrations (Menargues Marcilla *et al.*, 2012).

In this study, we investigate how markers of stress and body condition in Myanmar elephants vary through the year, their relation to seasonal climatic changes and, most importantly, how, at the population level, such measures relate to mortality and fertility rates, the key variables determining demographic trends. We use a unique demographic data set covering nearly a century of the world's largest semi-captive Asian elephant population, combined with longitudinal measures of stress and body condition. We determine monthly variation in faecal glucocorticoid metabolite concentrations and individual body weight across a typical entire monsoon cycle, and investigate their association with climatic variation and seasonality of averages of births and deaths among captive-born timber elephants between 1944 and 2000. We use such historic data because the slow life history of Asian elephants requires several decades of data to determine the average birth and death rates. We also investigate differences between sexes and age groups, given that males and females may have different baseline levels of stress hormones as well as different seasonal patterns in their excretion (Kudielka and Kirschbaum, 2005), and GCM concentrations have been shown to vary with age in many species, including elephants (Brown *et al.*, 2010). We illustrate the importance of combining ecological, physiological and demographic data into a comprehensive picture of the factors influencing survival and reproduction, an approach that could be critical in conservation studies.

## Materials and methods

### Study population

The Extraction Department of Myanma Timber Enterprise (MTE) collects and maintains records of all individual elephants owned by the state. This comprehensive countrywide system is unique to Myanmar and is the equivalent to studbooks kept by Western zoos. Data for each individual include registration number, sex, maternal identity, birth and death dates, location of birth, origin (wild caught or captive born), capture method (if applicable), year of capture, age of taming, birth dates, identities and cause of death (if applicable) of offspring. Births of captive-born elephants are recorded precisely, while an estimated date of birth is assigned to wild-caught elephants. In order to check the health and working ability of each elephant, individual logbooks are updated on a bi-monthly basis. The multiple sources of data recorded by the MTE (individual elephant logbooks, annual extraction reports and end-of-the-year reports from each region) allow effective cross-checking of any apparent errors.

For all elephants kept by MTE, workload and rest periods are seasonal and defined by law; all state-owned elephants are subject to the same central government regulations for hours of work. For example, in 2010 all mature elephants (17–55 years old) worked 3–5 days a week (depending on weather and forage availability), 5–6 h a day (maximum 8 h), with a break at noon. Work primarily involves pushing and dragging logs, which are selectively felled (Toke Gale, 1974). In 2002, half of logs felled in Myanmar involved the use of elephants for extraction (Shepherd, 2002).

Throughout the year, Myanmar timber elephants forage at will and experience very little supplementary feeding. Therefore, the seasonal variation in body weight could more closely follow resource quality and climatic conditions than in other captive populations (Toke Gale, 1974). There is no selective breeding, and most reproduction takes place at night in the forest, where both captive and wild bulls (depending on location) have access to oestrus females (Mar, 2007). For this reason, timber elephants are characterized as semi-captive. Weaning age is ∼4 years, and at ∼5 years the calves are separated from the maternal herd, tamed and given a name, an identification number and a mahout (individual caretaker and rider). Calves are then trained and used for light work duties until they are put in the workforce at the age of 17 years as mature logging elephants, continuing to work until retirement at the age of 55 years (Mar, 2007).

### Climatic and demographic data

**Figure 1:**
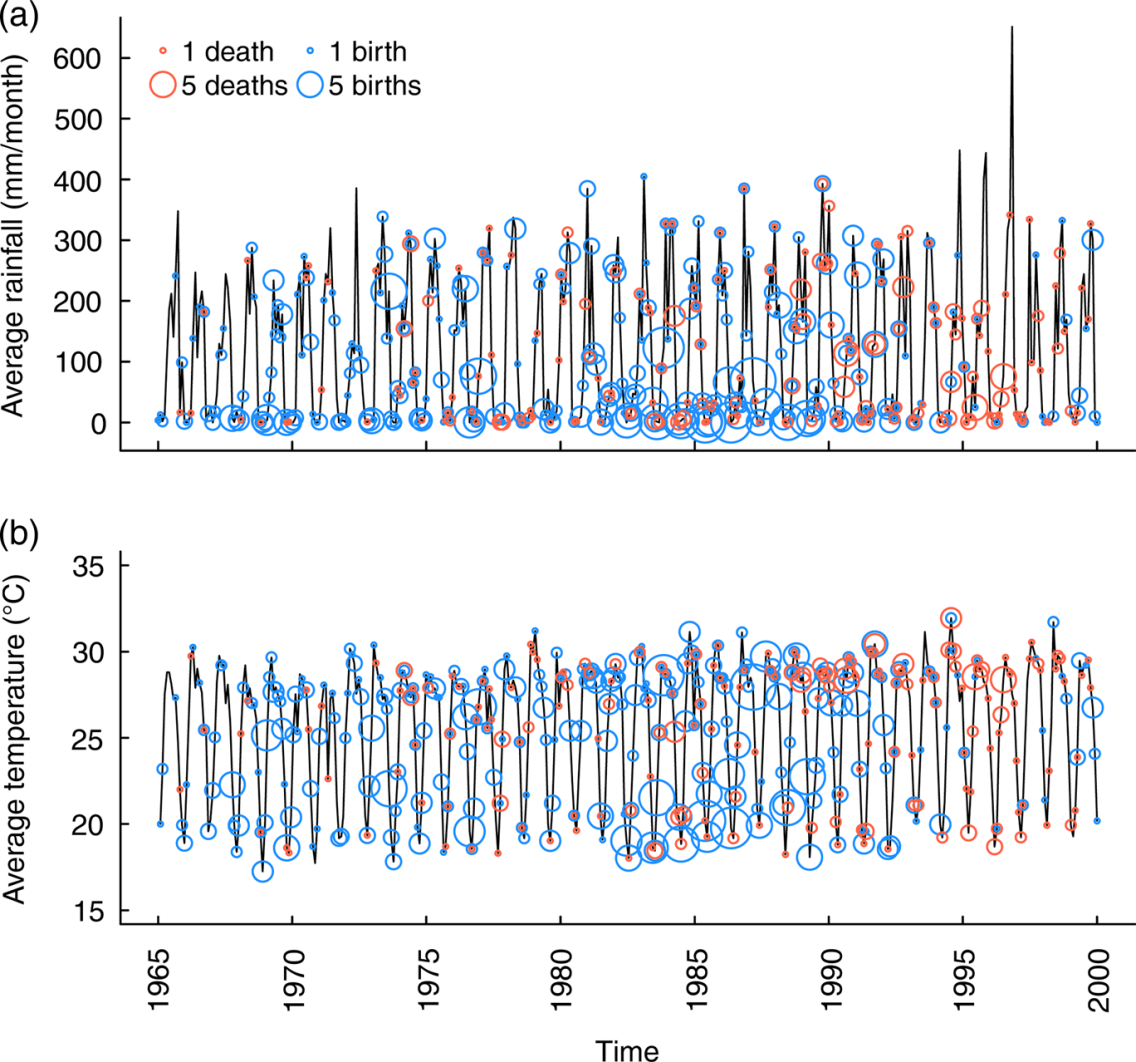
Consistent annual seasonality of climate, births and deaths over 35 years in Myanmar elephants in the years 1965–2000. Climate is displayed as black curves showing rainfall (in millimetres per month; (**a**) and temperature (as mean monthly temperature; (**b**). The circles show the timing of births in blue (*n* = 1024) and deaths in red (*n* = 315), with the diameter of the circles proportional to the number of events in each month. A total of 176 100 elephant-month observations are included.

Data on monthly average temperature (in degrees Celsius) and total rainfall (in millimetres) were available from the Department of Meteorology and Hydrology of Myanmar. The data cover four regions and the years 1965–2000. As there was little between-year variation (Mumby *et al*., 2013b), we used mean values across years and regions to obtain a temperature and rainfall value for each calendar month (Fig. 1). Monthly average temperatures varied from the extremes of 14–34°C (25 ± 3.7°C, mean ± SD), and monthly total rainfall varied from 0 to 906 mm (111 ± 102.6 mm, mean ± SD) in a highly consistent seasonal pattern. Temperatures peak in April and May, immediately before the onset of monsoon rains (June to October), and minima are observed from October to January, overlapping partly with the dry season (December to May), again following the same seasonal pattern over the 35 year study period (Fig. 1).

Demographic data are based on individual logbooks from 2350 elephants born in captivity with confirmed birth dates from the years 1944–2000 (Mumby *et al*., 2013a). Death dates are known for the 1040 of the 2350 captive-born individuals in the demographic data set that died by the end of the study. Both deaths and births are highly seasonal in this population, with deaths occurring largely in the dry months and at extremes of temperature, and conceptions peaking in the hot and dry months (February to May) across the entire study period (Fig. 1). Over the time period that the climatic and demographic data cover, elephants received treatments, such as prophylaxis for parasitic infection, and elephant management practices were modified, for example with the decreasing emphasis on capturing wild elephants. While these changes took place, they appear not to affect the general seasonally varying demographic patterns, which are consistent over time (Mumby *et al.*, 2013b).

### Weight and faecal sample data collection

We collected weight and faecal sample data for hormone analysis on a monthly basis for 12 months from 75 MTE elephants at two camps in Pyinmana, Mandalay region, from December 2011 to November 2012. All data collection was covered by ethical guidelines of the University of Sheffield. Our sampling period to characterize the monthly variation in body weight and markers of stress involved only 1 year (rather than decades as available for climate and demographic outcomes; see above); nonetheless, the patterns of climate (rainfall and temperature) and demography (births and deaths) have very clear seasonal patterns in this population, and these are stable across years (Fig. 1). Body condition and stress are therefore also likely to vary consistently between years. We confirmed that the climate during the year when we collected weight and faecal data followed a typical monsoon pattern using data from the Mai Hong Song weather station located close to the Myanmar border in Thailand (19.3°N, 97.833°E and elevation of 269 m; National Climatic Data Center, 2012). These data indicate a range in mean monthly temperature from 22 (January) to 30°C (April) and maximal rainfall of 325 mm in July. The period from December 2011 to November 2012 thus represents a typical year in this climate system against the long-term variation documented for climate, birth and death rates in our study population (Supplementary Fig. S1).

The sample consisted of 43 females and 32 males (39 captive-born and 36 wild-caught individuals), ranging in age from 4 to 69 years, with a mean age of 37 years. For analyses, these animals were categorized into the following three levels: under 17 years, including infants and juvenile elephants; 17–44 years, including prime-age working elephants undertaking the heaviest workload; and 45 years and over, which includes working elephants past the prime age and retired individuals (see Table 1 for age class distribution across months). Simultaneously with the faecal sample collection, we took monthly body measurements of weight of the same individuals using an Eziweigh 3000^®^ scale capable of weighing up to 9000 kg to the nearest 10 kg.

**Table 1:** Number of elephants measured each month by age class for glucocorticoid metabolite concentration (**a**) and body weight (**b**)

(a) Glucocorticoid metabolite concentration			
Month	Age ≤16 years	Age 17–44 years	Age ≥45 years
January	6	48	17
February	5	43	15
March	4	41	16
April	7	48	17
May	5	35	14
June	5	38	15
July	4	36	12
August	4	36	9
September	4	35	9
October	3	37	9
November	4	39	12
December	6	45	16
(b) Body weight			
Month	Age 17–44	Age 45 and older	
January	16	11	
February	65	38	
March	18	21	
April	27	21	
May	60	26	
June	49	17	
July	37	13	
August	–	–	
September	38	11	
October	37	11	
November	–	–	
December	52	27	

Elephants in (a) are all from Pyinmana, while (b) also includes elephants from Kawlin, East Katha and West Katha.

We restricted the weight measurements to non-pregnant adult elephants without very young calves. This ensures that the seasonal patterns captured reflect changes in the environment, rather than effects of growth or pregnancy; elephants in the young age category were still growing rapidly, and weight gain in pregnant females may be because of pregnancy rather than changes in condition. Pregnant females may also exhibit patterns of stress hormone changes associated with pregnancy (Kajaysri and Nokkaew, 2014) rather than the seasonal variation in the environment we aim to study. Our aim is to focus on variation in markers of body condition and stress, rather than already pregnant ones. The shoulder height of these adults did not change throughout the year, and therefore any changes in weight represent changes in body condition rather than growth (ANOVA for height by month, *F* = 0.019, *P* = 0.89). Given that body weight measurements for these individuals were available for only 7 of the 12 months, we supplemented the body weight data with similar longitudinal measures of 51 non-pregnant adult elephants from three other camps in Kawlin (*n* = 24; 19 females and five males), East Katha (*n* = 15; eight females and seven males) and West Katha (*n* = 12; 10 females and two males). Although the management and work schedules are the same across camps, we also statistically controlled for variation by camp (see ‘*Statistical analysis*’) to ensure that these supplementary elephants could be included in analyses. This gave a total of 10 months of body weight measurements (Table 1), with only August and November missing, and including individuals followed for 1–7 months (mean = 5 months ± SD 1.7).

### Endocrine analysis

We collected faecal samples for endocrine analyses as soon as possible after defecation in the morning and stored them in ziplock sandwich bags at −20°C until drying in a hot air oven at 50°C. Using dry samples and measuring glucocorticoid metabolitcs per gram of dry faeces has been shown to reduce the effects of seasonal changes in diet on hormone-excretion rates (Wasser *et al*., 1993). Dry faeces were gently separated from plant material, and hormones were extracted from faecal material using a validated protocol for Asian elephants (Watson *et al.*, 2013). The sensitivity of the assay was 0.03 ng/ml. The interassay coefficient of variation (CV) for the high-concentration control was 15.1% and for the low-concentration control was 9.4%, whilst the intra-assay CV was 9.71% for the high-concentration control and 8.93% for the low-concentration control. The corticosterone enzyme immunoassay was validated for Myanmar elephant faeces by demonstrating parallelism between serially diluted samples and the respective standard curve, and 90% recovery of added standard hormone to pooled samples (Brown *et al.*, 2004).

### Statistical analysis

#### Seasonal variance in glucocorticoid metabolite concentrations and body weight

To test for monthly variation in glucocorticoid metabolite concentrations (in nanograms per gram of dried faeces), we implemented a linear mixed model using the package ‘nlme’ (Pinheiro *et al.*, 2013) in R version 3.0.2 (R Core Development Team, 2011). We included the identification number of each elephant as a random effect in the model to account for the dependency between different measurements of the same individuals, and month (factor with 12 levels), age group (factor with three levels) and sex (binary) as fixed effects. We accounted for temporal autocorrelation, i.e. measurements from consecutive months being similar purely because they were recorded closest to each other in time, in all our seasonal models (Haddad, 2004). We used the function corARMA for the package nlme and selected the correlation structure resulting in the model with the lowest Akaike information criterion (AIC). We implemented similar linear mixed models to test for seasonal variation in body weight, with the following modifications: two age groups were used (non-adults were not included); month was described by 10 levels (no data were collected in August and November); and an additional fixed effect for location (factor with four levels) was included to take into account the different camps in which weight measurements were taken.

To test for whether within individual variation by month of GCM also occurred, we created a new variable of within-individual centred GCM values, which is an individual's mean GCM over all months minus the value for each month, following van de Pol and Wright (2009). We then ran a model with this term as the outcome variable and a further term of individual mean GCM to control for their average values. We repeated these models with within-individual centred weight as the response variable and an additional term to control for location (factor with four levels) to test for individual-level monthly variation in weight.

For both GCM and weight, interactions between age group and month and sex and month, to test whether the seasonal patterns differ between animals of different ages or sex, are not included in the final models presented here. When tested, these were non-significant, but because of the large number of degrees of freedom required to test them, this is not conclusive evidence for lack of differences in pattern across months by sex and age group. As month had an effect in both models for GCM and weight (see Results), we then computed average GCM values and average weight in each month independently for each sex–age group combination for the following analyses, which are performed at the population level. In all analyses, *P*-values <0.05 were referred as being ‘significant’ for simplicity.

#### Correlation between glucocorticoid metabolite concentration and body weight

To test for differences in the seasonal pattern of faecal GCM and weight, we conducted a cross-correlation analysis. Specifically, we measured the correlation between time series established for faecal GCM and weight for all possible time shifts in months and selected the time shift that corresponded to the maximal correlation value. The correlations computed were non-parametric Spearman correlations. The obtained correlation coefficient, ρ, varies between ρ = −1 (seasonal patterns being in perfect opposition in the two variables) and ρ = +1 (seasonal patterns in perfect synchronicity), via 0 (patterns strictly independent). We obtained GCM and weight input values for each month from the mean values outlined above. We allowed both positive and negative time lags between the traits, because we had no *a priori* hypothesis to indicate which one would precede the other.

#### Correlation of glucocorticoid metabolites and body weight with climatic and demographic variables

We repeated the cross-correlation analyses to test whether GCM concentrations and weight correlated in time with the following variables known to vary seasonally in this population: number of deaths by month; number of births by month; total monthly rainfall (in millimetres); and mean monthly temperature (in degrees Celsius). For the demographic parameters, we assumed that death would follow in time the measurements of stress and condition (GCM concentration and weight) and therefore we allowed it to have only 0 or negative time lags in comparison to GCM and weight. For births, we allowed negative and positive time lags to take into account that births also represent the timing of conception (22 months prior to birth); a +2 time lag could represent a correlation between GCM or weight and timing of conception. As the climatic variables were expected to influence the measures of stress and condition, we allowed them to have 0 or positive lags in comparison to GCM and weight.

## Results

### Seasonal variation in glucocorticoid metabolite concentrations

**Figure 2:**
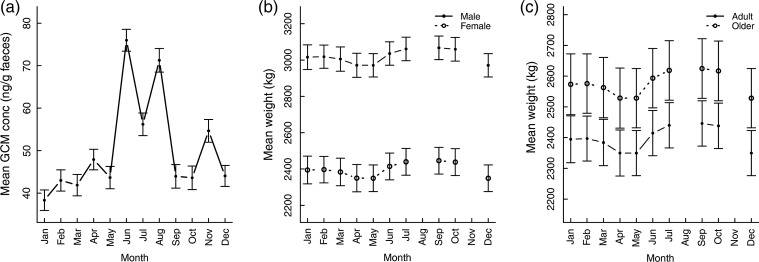
Monthly glucocorticoid metabolite concentration (**a**) and monthly weight by sex (**b**) and age group (**c**) in a population of Myanmar elephants [*n* = 70 for glucocorticoid metabolite (GCM) and *n* = 116 for weight]. Values for (a) are for adult males, for (b) are adults in the Pyinmana camp and for (c) are females in the Pyinmana camp. Juveniles are not included in the weight analysis because their weight increases rapidly through body growth.

We found substantial seasonal variation in mean monthly GCM concentrations, suggesting that the stress levels of a given elephant varied considerably between different seasons of the year, reaching their lowest in January and highest in June (*F* for factor month = 32.50, d.f. = 11, *P* ≤ 0.0001; Fig. 2a). The GCM concentration was significantly higher than the January reference group in June, July and August, in the monsoon season, and November, in the cool season (Supplementary Table S1). This pattern occurred across age classes and in both sexes (Supplementary Table S1); there were no significant differences in overall GCM concentrations by age group (*F* = 1.25, d.f. = 2, *P* = 0.29) and sex (*F* = 0.17, d.f. = 1, *P* = 0.57). The random term for individual identification indicates that there was large variation between individuals in their overall GCM concentrations. However, when we repeated these models on the individual level, with within-individual centred values for GCM each month as the outcome, the same seasonal pattern remained (Supplementary Table S2). Following this, we used the population-level patterns to investigate correlations between seasonal variation in GCM and other demographic and environmental characteristics.

### Seasonal variation in weight

We found that weight, too, varies by month, with highest weight overall in the monsoon months of July to October and lowest overall in the dry months of March and April (*F* for month as a factor = 6.53, d.f. = 9, *P* ≤ 0.0001; Supplementary Fig. S2). There were significant differences in weight by sex (*F* = 72.02, d.f. = 1, *P* ≤ 0.0001; Fig. 2b); for example, with adult males being on average 25.9% heavier than females in the January reference category. There were also significant differences by age group (*F* = 23.95, d.f. = 1, *P* ≤ 0.0001), with elephants in the oldest age class having higher body weight than their adult counterparts (Fig. 2c); for example, females in the oldest age class were on average 7.5% heavier than adult females. There was low between-individual variation in body weight (see Supplementary Table S3), which is expected because the body size of elephants is constrained within certain morphological limits and can vary only by a certain proportion (Sukumar *et al.*, 1988), unlike glucocorticoids, which could potentially double between months. When we repeated these models on the individual level, with within-individual centred values for weight each month as the outcome, the same seasonal pattern remained (Supplementary Table S4). Following this, we used the population-level patterns to investigate correlations between seasonal variation in weight and other demographic and environmental characteristics.

### Correlation of glucocorticoid metabolite concentration and body weight with climatic and demographic variables

#### Cross-correlation of glucocorticoid metabolite concentration and body weight

We tested the cross-correlation between the mean values for GCM concentration and mean observed weight by month to determine whether the two measures of stress and condition followed similar seasonal patterns and whether this pattern involved any time lags. The strongest correlation was when body weight was lagged by 3 months following GCM measurement (Supplementary Fig. S2; correlation ρ = 0.69, *P* = 0.035; for significance of cross-correlation see Supplementary Fig. S3). The fact that the two markers of stress and condition were correlated positively, with maximal weight occurring 3 months following maximal GCM, was interesting because we initially expected a negative correlation. However, body weight and GCM may respond differently to environmental conditions and they may not be solely markers of stress, but may also reflect other aspects of the animal's health. Therefore, in the investigation between these physiological markers and demographic and environmental variables, weight and GCM were considered separately.

#### Cross-correlation of climate, stress and body condition markers

**Figure 3:**
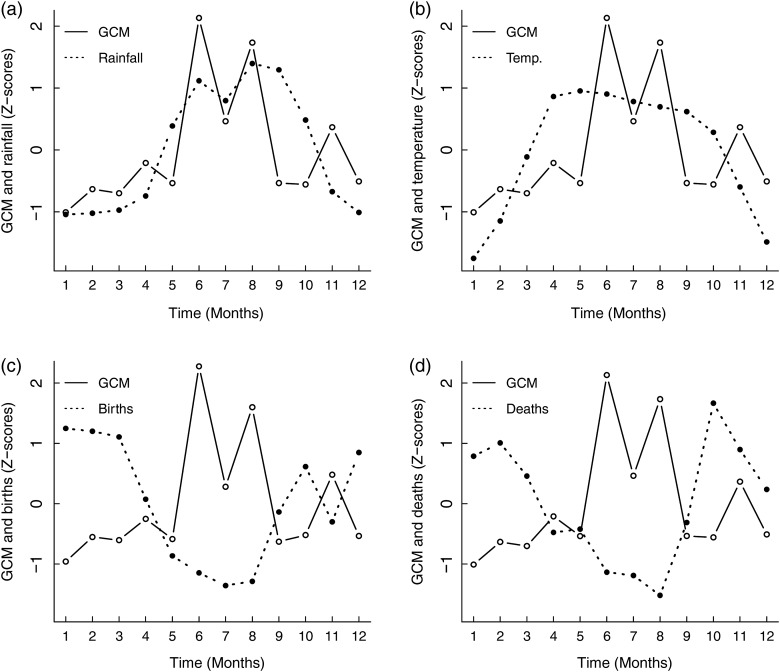
Cross-correlations between glucocorticoid metabolite (GCM) concentration andtotal monthly rainfall with no time lag (**a**), average monthly temperature with no time lag (**b**), number of births with no time lag (**c**) and number of deaths with a time lag of 3 months (**d**) in a population of Myanmar elephants. The GCM values for (a), (b) and (d) are mean values for the whole population, controlling for age and sex. The GCM values for (c) are for adult females (aged >16 years). Values are expressed as Z-scores.

We then investigated whether climatic variation or the human-induced seasonal work pattern coincided with monthly variation in GCM concentration and body weight or were correlated with monthly birth and death rates. Intriguingly, although body weight peaked during the period with the highest rainfall and best food availability, so did the GCM concentration, indicating potentially higher stress levels. The values for GCM concentration in each month were positively correlated with total monthly rainfall (averaged across four regions for the years 1965–2000) in the same month and peaked during monsoon (ρ = 0.69, *P* = 0.017; Fig. 3a); for example, the mean value for GCM concentration was 83.6% higher during the month with the highest rainfall (August) in comparison to the month with the lowest rainfall (January). The GCM concentration was also correlated with mean monthly temperature, with the strongest correlation being between temperature and GCM in the same month (ρ = 0.49; Fig. 3b); however, this correlation did not reach statistical significance (*P* = 0.067; see Supplementary Fig. S4b).

**Figure 4:**
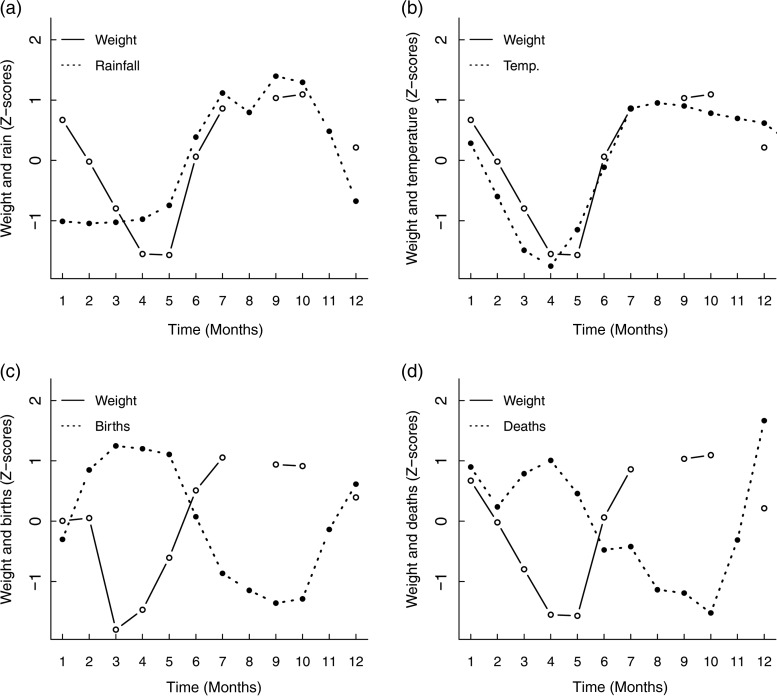
Cross-correlations between body weight and total monthly rainfall with a time lag of 1 month (**a**), average monthly temperature with a time lag of 2 months (**b**), number of births 2 months previously (**c**) and number of deaths with a time lag of 1 month (**d**) in a population of Myanmar elephants. Weight values for (a), (b) and (d) are mean values for the whole population, controlling for age and sex. Weight values for (c) are for adult females (aged >16 years). Values are expressed as Z-scores.

Likewise, values for body weight had a positive correlation with total monthly rainfall across age groups, sexes and birth origins. The increase in weight followed rainfall with a time lag of 1 month (ρ = 0.79, *P* < 0.05; Fig. 4a and Supplementary Fig. S5a); for example, female adult elephants were 4.9% heavier in the month following the highest rainfall month (August) in comparison to the month after the lowest rainfall (January). Body weight followed the peak in temperature with a time lag of 3 months (*ρ* = 0.79, *P* < 0.05; Fig. 4b), and the correlation was statistically significant (Supplementary Fig. S5b).

#### Cross-correlation of glucocorticoid metabolite concentration, birth and death rates

Although elephants are capable of reproducing throughout the year, their birth rate varied by a factor of 1.8 between the highest month (January) and lowest month (July). Our results indicate that increases in stress levels may be associated with population birth rate (*n*_births_ = 2350 for the years 1948–2000); GCM concentrations among females of reproductive age showed a significant inverse correlation with the proportion of births in the same month (ρ = −0.69, *P* = 0.016; Fig. 3c); for example, GCM concentration of prime-aged females was 37.1% lower during the month with the highest number of births (January) in comparison to the month with the fewest births (July, close to the start of the work season in June). Values for adult female weight showed a strong negative correlation with the number of births 1 month before (ρ = −0.79, *P* < 0.05; Fig. 4c; for time lags see Supplementary Fig. S5c). As the duration of gestation in elephants is 20–22 months (Lueders *et al*., 2012), the positive 1 month lag is close to the month of conception. Therefore, against our expectations, the negative correlation could represent a correlation between poor body condition in females of reproductive age and the peak in conceptions.

Death rate, too, exhibited seasonal variation, differing by a factor of 2.0 between the months with the highest and lowest mortality (January in the cool season and November at the end of the monsoon, respectively). The trough in deaths was correlated with the peak in GCM with a time lag of 3 months (ρ = −0.62, *P* < 0.05; see Fig. 3d; Supplementary Fig. S4). A strong negative correlation between death rate and body weight was also found, with the strongest negative correlation between body weight and deaths 1 month later (ρ = −0.62, *P* < 0.05; Fig. 4d; for significance see Supplementary Fig. S5d); the month with highest body weight (October) was 1 month before the month with lowest death rate.

## Discussion

We used longitudinal population-level data to link ecological variation, physiological markers and demography in endangered Asian elephants. While only a prospective study design with appropriate experimental manipulation of body condition and stress would allow the determination of causal individual-level effects of stress and body condition on survival and reproduction, we did uncover significant population-level correlations. Our main finding is the existence of significant correlations between seasonally varying markers of body condition and stress (body weight and GCM concentration; see Supplementary Fig. S4) and birth rates at the population level, the key variables determining demographic patterns in a population. We show that body weight peaks at the middle of the monsoon (between the months of July and October), while stress levels increase at the start of the monsoon and work season between themonths of June and August. More generally, our results indicate that shorter term seasonal variation in physiological characteristics at the individual level may be associated with larger scale variation in demographic and environmental conditions.

We showed that both GCM concentration and weight exhibited seasonal variation in working Asian elephants, with both variables peaking in the monsoon months of June to October. However, weight variation was opposite to the expected pattern, with higher body weights (i.e. superior body condition) recorded in the same months (or following a 1 month time lag) as higher mean GCM concentrations (i.e. higher stress hormone levels). This is in contrast to studies that have found a negative association between weight and cortisol in wild animals (Clinchy *et al.*, 2004; George *et al.*, 2014). A potential cause of this difference could be that the additional element of life in captivity, such as seasonal workload patterns, is affecting the association, for example by elephants working hardest in the months when they have best body condition and highest GCM. This is particularly interesting given the association between increased circulating glucocorticoids and inhibition of protein synthesis, meaning that high glucocorticoids can be associated with reduced muscle mass (Schakman *et al.*, 2013). Furthermore, there are examples of positive correlations between cortisol and body mass in captive birds (Heath *et al.*, 1998; Fokidis *et al.*, 2011) and wild rodents (Bauer *et al.*, 2013). There is also a possibility, as with all studies on faecal hormonal metabolites, that they do not reflect changes in serum cortisol (Fanson *et al.*, 2013), but another factor, such as diet. In our study, we measured GCM per gram of dried faeces to reduce such effects of seasonal changes in diet, as also done in other studies (e.g. Foley *et al.*, 2001).

Such seasonal variation in GCM and body weight is relevant, because both variables in this sample were negatively correlated with the number of births in the whole population. Although we can determine only a correlative association, if such relations indeed have a causal basis, then efforts to reduce female hormonal markers of stress during the working season through lower workload and efforts to increase individual body weight during the driest months, e.g. through supplementation of the most vulnerable age groups, may have the potential to improve birth and mortality rates of the world's largest semi-captive Asian elephant population. The type of supplementation is beyond the scope of this study, but increasing both total calories and specific nutrients may be applicable (Hatt and Clauss, 2006).

The GCM concentrations were highest in the months of June, July and August. These months correspond to the start of the monsoon season. Elephants end their annual rest period in June each year and begin working on tasks such as pushing logs to the rivers for transport (Mar, 2007). The shift from the rest period to the intense work season could potentially be associated with this increase in GCM concentrations. Given that the pattern in GCM mirrors the annual pattern in births in the same month, both patterns may be reflecting the seasonal workload schedule experienced by timber elephants. The pattern of deaths is also negatively correlated with GCM, but the time lag is by 3 months, meaning that it is less clear how the two are related. It is possible that the deaths reflect more closely the immediate climatic conditions, which are known to be associated with mortality in this population, rather than reflecting population-level patterns in a marker of stress. Alternatively, the relationship between GCM and pattern of deaths may be associated with immune function and disease, processes that may not act immediately, but might occur during that 3 month lag time to contribute to debility and eventual death, as has been found in human epidemiological studies (Maunder, 2005).

Studies of wild Asian elephants have shown seasonal variation in conception rate (Ishwaran, 1993; de Silva *et al.*, 2013). Hence, factors common to semi-captive and wild elephants may influence the timing of conception rather than (or in addition to) workload. For example, a study on elephants in Sri Lanka found a peak in conceptions at the end of the dry season (de Silva *et al.*, 2013), although this pattern does not appear to be consistent across all Sri Lankan elephant populations in all years (Ishwaran, 1993). We also note that GCM concentrations were not elevated in all working months, indicating that the elephants may be responding normally to glucocorticoids and the elevated levels could be associated with intense workload and do not appear to represent chronic stress, which would be a major welfare concern. Experimental manipulations of work schedules could be used to test this possibility. Alternatively or additionally, a high joint temperature–humidity index in the monsoon months could cause elevated glucocorticoids in the elephants. Elephants have a low surface area-to-volume ratio and could be prone to overheating in these months, causing elevated adrenal activity (Weissenböck *et al.*, 2011). This explanation has been proposed to explain the prolonged reproductive cycles observed in captive non-working Thai Asian elephant females in the monsoon season (Thitaram *et al.*, 2008), indicating that these conditions could affect other hormonal cycles, too. It is also noteworthy that although the seasonal pattern in GCM concentration indicates a peak in adrenalin activity in the months June to August, it then falls back to the concentrations recorded before the peak.

Overall, the values recorded are within the range observed in both Asian elephants in zoo settings (Laws *et al.*, 2007) and wild African elephants (Foley *et al.*, 2001), potentially indicating that the working life of the Myanmar timber elephants does not elevate the hormone levels beyond what is seen in these other groups of elephants. Although there are limitations to comparing values from different studies, and particularly different species (Lane, 2006), our results appear to be consistent with the idea that the transition from rest to intense work, and not whether the elephants work at all or not, could be associated with the elevated glucocorticoid concentrations in June to September. This association between activity and GCM concentration could be investigated further by measuring activity schedules in the elephants, for example using accelerometry (Soulsby *et al.*, 2011), and correlating these energy usage measures with GCM.

In contrast, body condition as measured by body weight remained increased from the rest season to July to October. Given that high weights and good body condition coincide with the wet monsoon months (and show a highly positive correlation with rain, with a 1 month time lag), the high body weights in the monsoon season could be reflecting the better availability and higher quality of forage in these months (Sukumar, 1989; Mar, 2007; Campos-Arceiz *et al.*, 2008), with a short delay that may be associated with the improved resources being converted to body fat stores. Our previous analyses (Mumby *et al*, 2013b) indicated an association between monthly survival rates and climatic conditions, with months characterized by intermediate temperatures and high rainfall having the highest survival rates. It appears that body weight could link the climatic and survival effects, given the association between high body weight and low mortality rates found in monsoon months. The 1 month time lag in the negative correlation of deaths and body weight could be reflecting the time it takes for poor body condition to affect mortality. For example, the proximate causes of low body weight, such as disease, undernutrition, malnutrition or overwork, may take time to influence mortality through either direct debility or indirect effects on immune function.

Our results have implications for the management of captive elephant populations, and highlight the potential impacts of environmental conditions on elephant body weight. As the heaviest working periods overlap with the months during which body weights are highest and climatic conditions are optimal for elephant survival, we suggest that the current pattern of work may maintain the work-related stress at a minimal level. If, as we propose, the transition from rest period to work period does cause a peak in GCMs that lasts up to 3 months, a management solution could be to transition slowly into work, with reduced hours in the first month of work. Regarding the young working elephants, training currently takes place over as little as 4 weeks (Min-Oo, 2010), and a less intensive training regimen could reduce the seasonal variation in stress in these individuals. We suggest that this could be important both for the welfare of the elephants and to ensure that they are capable of work, because high GCM concentrations are associated with suppressed immune function (Mason, 2010).

Understanding the physiological correlates of increased mortality or low birth rate will help to direct better interventions. The greatest threat for contemporary Asian elephant populations is low birth and survival rates; sustainable captive populations are currently unattainable. Populations of wild elephants maintain high rates of annual age-specific fecundity; for example, rates reach 0.20–0.25 offspring per female between the ages of 15 and 45 years in Amboseli, Kenya (Moss, 2001) and are on average between 0.13 and 0.17 offspring per female in wild Sri Lankan elephants (de Silva *et al.*, 2013). However, rates in semi-captive populations are much lower. In Tamilnadu and Karnataka of India in the early 20th century, the annual fecundity rate of captive elephants in forest camps was about 0.1 per adult female, and then rose between 1969 and 1989 to 0.16 per adult female (Sukumar, 2003). As a result of the relatively high survival in these populations in the past, the populations could maintain a low growth rate. In Myanmar, the fecundity rates are around 0.1 per adult female on average (Robinson *et al.*, 2012; Hayward *et al.*, 2014; Lahdenperä *et al.*, 2014), and the survival rates are also lower than in the populations in India; therefore, small changes to affect survivorship or breeding could have a relatively large impact on the population sustainability. In contrast, elephants in zoos have been shown to have annual fecundity rates consistently below 0.05 per adult female (Clubb *et al.*, 2009), making sustainability a much more difficult prospect.

Low birth rates may be associated with high stress levels associated with maternal workload, management practices and access to mates. We show that GCM concentration has a strong negative association with birth rates even when body condition is highest, and that GCM concentration peaks in the monsoon months at the beginning of the work season. A potential solution, therefore, would be to reduce the workload of selected females of prime reproductive age and to allow these females better access to males for mating. This could increase the birth rate in the months in which it is currently low; if birth rates in the peak months of December to March were maintained throughout the year, the result would be an increase in births by 20%. There are some inherent risks, such as potential for competitive interactions between females and stress associated with presence of males (Beehner *et al.*, 2005). However, given that the population of timber elephants represents the largest semi-captive population of Asian elephants, solutions to improve its viability contribute towards maintenance of a genetically diverse pool of elephants in Southeast Asia. More generally, our results point to the potential consequences of variation in body condition and stress, which may also be seen in wild populations when resources are depleted by habitat loss and fragmentation.

Our study highlights the importance of looking at several population-level outcomes in populations simultaneously, as well as their underlying ecological drivers. For example, we found that GCM and body weight were not inversely correlated, as often assumed, and the same relation between markers of body condition and stress may occur in other species. This is particularly important in this working population, which faces seasonal changes in work as well as climate and resource availability. We also demonstrate that individual variation in condition or stress may parallel large-scale patterns in mortality and fertility of populations over time in a long-lived and continuously breeding species.

## Supplementary material

Supplementary material is available at *Conservation Physiology* online.

## Funding

V.L., H.S.M. and K.U.M. would like to thank the Leverhulme Trust, Natural Environment Research Council for funding the Myanmar Timber Elephant project. The project received additional support from the Nando Peretti Foundation (V.L.), The Rufford Foundation (K.U.M.) and European Research Council (V.L.).

## Supplementary Material

Supplementary Data
