# Supplementary material for: Stress and body condition are associated with climate and demography in Asian elephants
Source: Conserv Physiol. 2015 Jun 30;3(1):cov030. doi: 10.1093/conphys/cov030 (PMC4778474; doi:10.1093/conphys/cov030)
Supplement: Supplementary Data [file cov030supp.zip › cov030supp.pdf]

Supplementary Materials

The following Supporting Information is available for this article online

Table S1 Coefficients for a GLMM of glucocorticoid metabolite concentration by month over a year in 70 elephants from Myanmar. The baseline is a female in the prime adult (age 17-44 years) age category. A term to control for autocorrelation between months was used.

Fixed effects	Value	Std.Error	df	t-value	p-value
(Intercept)	39.306	2.070	615	18.992	<0.001
February	4.650	2.547	615	1.825	0.068
March	3.549	2.545	615	1.395	0.164
April	9.562	2.554	615	3.743	<0.001
May	5.326	2.738	615	1.945	0.052
June	37.632	2.658	615	14.159	<0.001
July	17.850	2.856	615	6.250	<0.001
August	32.910	2.927	615	11.245	<0.001
September	5.622	2.945	615	1.909	0.057
October	5.264	2.937	615	1.793	0.074
November	16.319	2.839	615	5.748	<0.001
December	5.712	2.504	615	2.281	0.023
Old age class	2.018	1.910	69	1.057	0.294
Youngest age class	-2.224	3.009	69	-0.739	0.462
Male	-0.953	1.670	69	-0.571	0.570
Random effect	Std.Dev	Residual			
ID	378.935	102.124			

Table S2 Coefficients for a GLMM of within-individual variation in glucocorticoid metabolite concentration by month over a year in 70 elephants from Myanmar. The model measures whether the within-individual value each month differs from the within-individual mean. The baseline is a female in the prime adult (age 17-44 years) age category. A term to control for autocorrelation between months was used.

Fixed effects	Value	Std.Error	df	t-value	p-value
(Intercept)	8.045	4.720	615	1.704	0.089
February	-4.256	2.530	615	-1.682	0.093
March	-2.886	2.553	615	-1.130	0.259
April	-9.566	2.445	615	-3.913	<0.001
May	-4.244	2.642	615	-1.606	0.109
June	-36.568	2.589	615	-14.125	<0.001
July	-16.799	2.669	615	-6.294	<0.001
August	-31.855	2.718	615	-11.722	<0.001
September	-4.867	2.733	615	-1.781	0.076
October	-4.921	2.717	615	-1.811	0.071
November	-15.879	2.627	615	-6.045	<0.001
December	-5.870	2.489	615	-2.358	0.019
Old age class	-0.227	1.350	68	-0.168	0.867
Youngest age class	-0.140	2.141	68	-0.065	0.948
Male	0.646	1.170	68	0.552	0.583
Within individual mean GCM	0.053	0.087	68	0.608	0.545
**Random effect**	**Std.Dev**	**Residual**			
ID	<0.001	14.615			

Table S3 Coefficients for a GLMM of body weight by month over one year in 116 elephants from Myanmar. The baseline is a female in the prime adult (age 17-44 years) age category in the Pyinmana camp. A term to control for autocorrelation in the data did not improve the model fit and was therefore not included.

Fixed effects	Value	Std.Error	df	t-value	p-value
(Intercept)	2408.806	65.685	460	36.672	<0.001
February	-7.303	26.633	460	-0.274	0.784
March	-15.759	29.456	460	-0.535	0.593
April	-44.988	27.662	460	-1.626	0.105
May	-54.615	27.159	460	-2.011	0.045
June	6.887	28.131	460	0.245	0.807
July	35.507	30.176	460	1.177	0.240
September	41.618	30.280	460	1.374	0.170
October	33.817	30.364	460	1.114	0.266
December	-52.032	27.608	460	-1.885	0.060
Old age class	158.794	81.114	107	1.958	0.053
Male	622.884	77.497	107	8.038	<0.001
East Katha	134.473	118.904	107	1.131	0.261
Kawlin	-168.534	94.135	107	-1.790	0.076
West Katha	-120.278	131.974	107	-0.911	0.364
**Random effect**	**Std.Dev**	**Residual**			
ID	0.0002	15.858			

Table S4 Coefficients for a GLMM of within-individual variation in body weight by month over one year in 116 elephants from Myanmar. The baseline is a female in the prime adult (age 17-44 years) age category in the Pyinmana camp. A term to control for autocorrelation in the data did not improve the model fit and was therefore not included.

Fixed effects	Value	Std.Error	df	t-value	p-value
(Intercept)	-5.396	34.663	460	-0.156	0.876
February	5.127	23.992	460	0.214	0.831
March	14.517	26.568	460	0.546	0.585
April	43.408	24.872	460	1.745	0.082
May	54.465	24.418	460	2.230	0.026
June	-5.599	25.340	460	-0.221	0.825
July	-32.834	27.102	460	-1.211	0.226
September	-37.875	27.173	460	-1.394	0.164
October	-29.883	27.242	460	-1.097	0.273
December	49.480	24.846	460	1.991	0.047
Old age class	-1.997	9.013	106	-0.222	0.825
Male	-0.824	10.465	106	-0.079	0.937
Within individual mean weight	0.001	0.010	106	0.117	0.907
East Katha	-18.431	16.143	106	-1.142	0.256
Kawlin	-25.237	14.532	106	-1.737	0.085
West Katha	-18.919	18.859	106	-1.003	0.318
**Random effect**	**Std.Dev**	**Residual**			
ID	0.004	94.134			

Figure S1

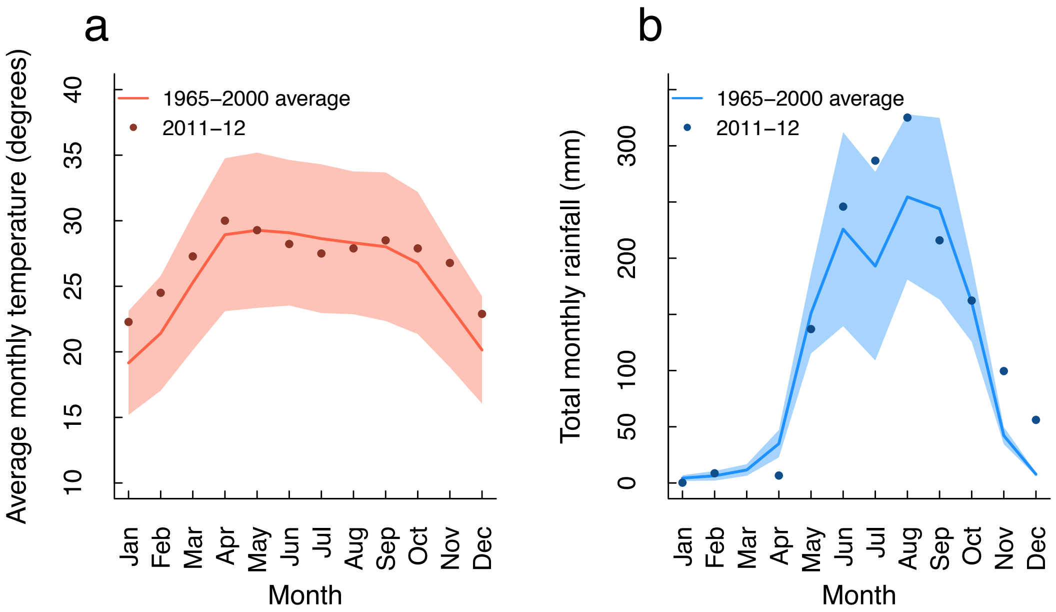

Climate in Myanmar from 1965-2000 and in the study year 2011-12 The lines indicate (a) Average temperature and (b) total rainfall observed in 4 timber elephant camps in Myanmar 1965-2000. The shaded areas show the standard deviation around the means. The points indicate the weather conditions from December 2011 to November 2012, when the GCM and weight study samples were collected.

Figure S2

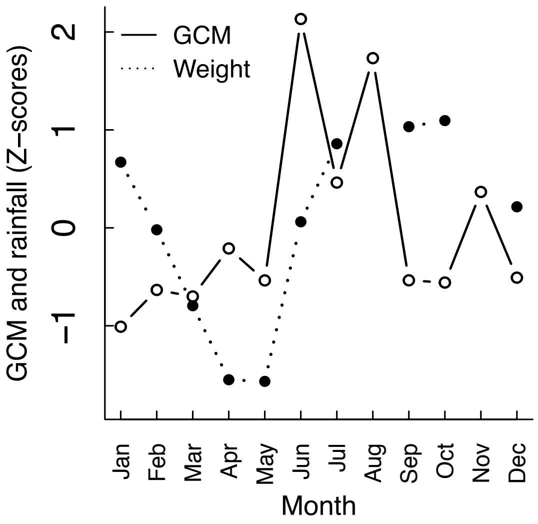

Seasonal variation in glucocorticoid metabolites and body weight in a population of Asian elephants. Points represent population means for both sexes and all age classes and error bars indicate standard errors around these means.

Figure S3

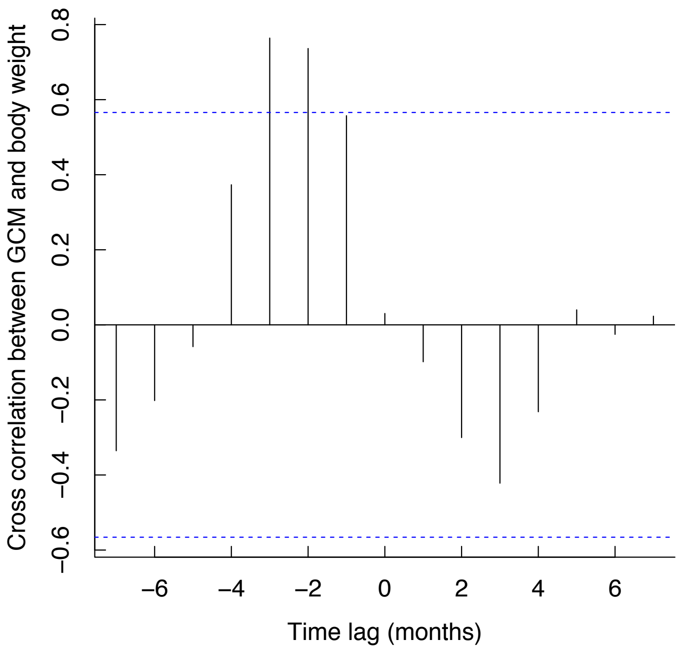

Correlation coefficients between mean glucocorticoid metabolite concentration (GCM) and monthly body weight at a range of time lags in a population of Myanmar elephants. The blue lines indicate the 95% confidence intervals; lines crossing the blue lines are considered to be statistically significant.

Figure S4

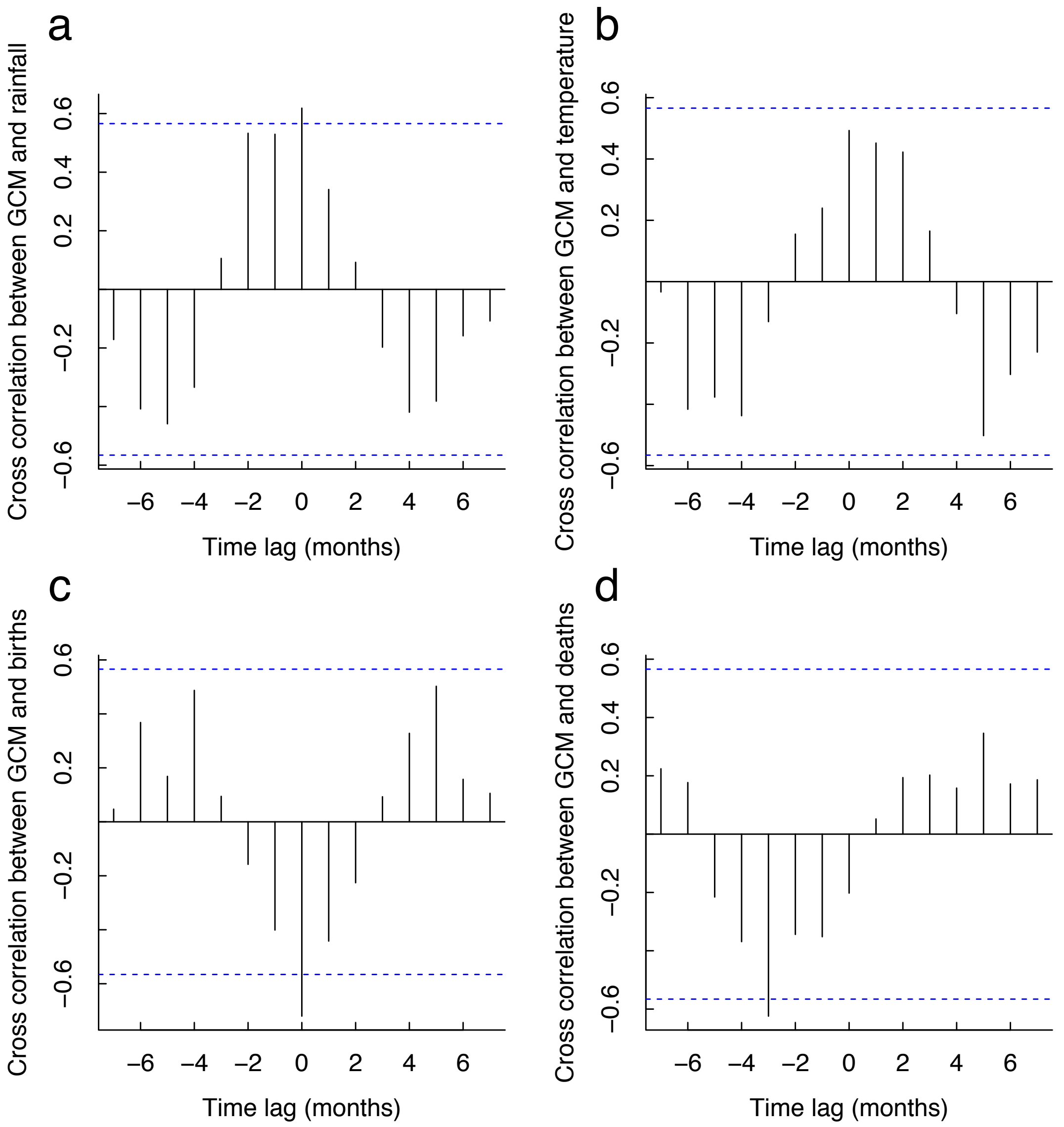

Correlation coefficients between mean glucocorticoid metabolite concentration (GCM) and (a) total rainfall, (b) mean temperature, (c) monthly births and (d) monthly deaths at a range of time lags in a population of Myanmar elephants. The blue lines indicate the 95% confidence intervals; lines crossing the blue lines are considered to be statistically significant. For (c) only GCM values of reproductive aged females (over 16) were considered.

Figure S5

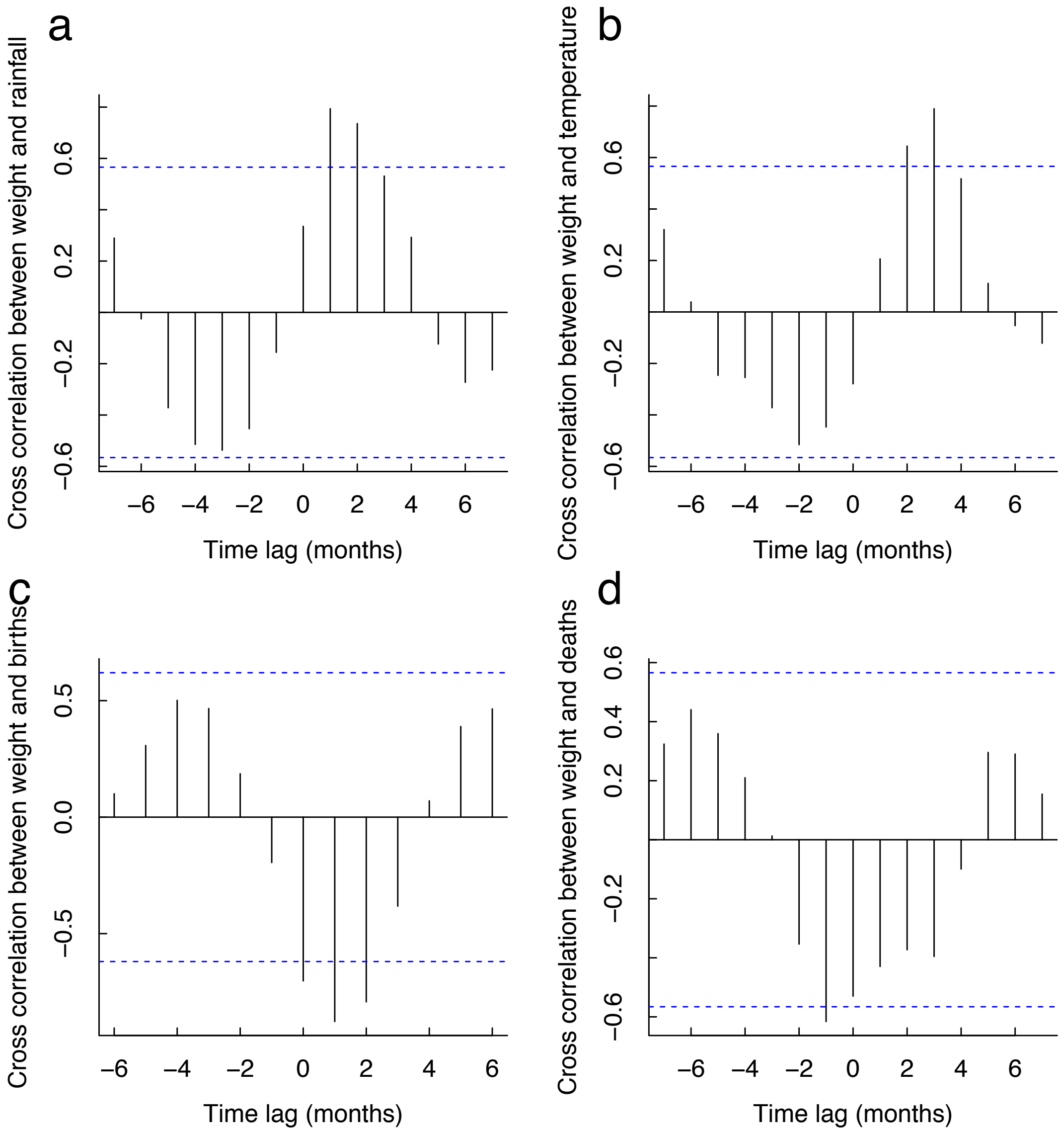

Correlation coefficients between mean body weight and (a) total rainfall, (b) mean temperature, (c) monthly births and (d) monthly deaths at a range of time lags in adult Myanmar elephants. The blue lines indicate the 95% confidence intervals; lines crossing the blue lines are considered to be statistically significant. For (c) only weight values of reproductive aged females (over 16) were considered.
